# Integrity Assessment of High-Performance PVC Pipes for Thermal Wells

**DOI:** 10.3390/polym15173593

**Published:** 2023-08-29

**Authors:** Sayyad Zahid Qamar, Tasneem Pervez, Farooq Al-Jahwari

**Affiliations:** Mechanical and Industrial Engineering Department, Sultan Qaboos University, Muscat 123, Oman; tasneem@squ.edu.om (T.P.); farooq@squ.edu.om (F.A.-J.)

**Keywords:** steam injection well, corrosion, high-strength PVC, PVC screen, saline water aging, compression testing, anisotropy

## Abstract

In wells with high-viscosity crude oil, steam injection is used to improve the fluidity of the heavy oil. Screens made of steel are employed for sand control, with corrosion being a serious problem. Non-metallic pipe materials, such as novel high-strength PVC, are therefore being tried out. This paper presents the results of an integrity assessment of large-diameter hard PVC pipes under compressive loading. Plain, built-up, and slotted pipes were subjected to a 3-month aging process in saline water. Strain gauge sets were used for dynamic testing of longitudinal and transverse deformations. Values of fracture strength, total deformation, and anisotropy (Poisson’s ratio) were extracted from the stress–strain graphs and analyzed. Upon aging in saline water, the stiffness of all pipes increased and was the highest for slotted pipes. Maximum stress after soaking was reduced by 11–12%. The ductility was the highest for plain pipes and the lowest for built-up pipes. Poisson’s ratio remained almost constant for all pipes and aging conditions. The good news for field applications is that overall, aging had only a minor impact on the major compressive properties. The main conclusion was that corrosion-prone steel pipes and sand screens can be successfully replaced by corrosion-free high-strength PVC pipes and screens for water transport applications in thermal wells. This work provides a scientific basis for the structural integrity assessment of this new PVC and helps field engineers in the proper pre-deployment selection of pipes for target oilfields.

## 1. Introduction

### 1.1. Thermal (Steam Injection) Wells

Steam injection wells are a type of enhanced oil recovery (EOR) method that uses steam to heat heavy oil and bitumen in the reservoir, making it easier to flow to production wells. Steam injection wells are typically used in oilfields that have high-viscosity oils, which are difficult to produce using conventional methods [1]. There are two main types of steam injection wells: cyclic steam stimulation (CSS) and steam flooding (SF). In CSS, steam is injected into a well for a period, followed by a period of production. This cycle is repeated several times until the oil recovery is maximized. In SF, steam is injected continuously into a well, while oil is produced from other wells. 

The steam injection process works by heating the oil in the reservoir, which reduces its viscosity. This makes it easier for the oil to flow through the reservoir and to the production wells. Steam also helps to break the oil down into smaller droplets, which makes it easier to extract. Though steam injection wells are a relatively expensive EOR method, they can be very effective in recovering heavy oil and bitumen. In some cases, steam injection can recover up to 50% of the oil in place [2].

The major advantages of steam injection wells are that they recover heavy oil and bitumen that is difficult to produce using conventional methods, can be very effective in increasing oil recovery, and are relatively environmentally friendly. Their disadvantages are that they are expensive to operate, require a large amount of water, and can damage the reservoir [3].

### 1.2. Nonmetallic Pipes in Oil Drilling

Increasing demand for oil is leading to longer drilling ranges during oil searches, including deep-water platforms and high-temperature land-based platforms [4]. Special nonmetallic materials (polymers and composites) are being developed to withstand these extreme conditions in oil wells [5]. Although nonmetallic pipes are costlier, their lightweight nature reduces installation costs. Over time, metal pipes face corrosion, requiring replacement or repair. Nonmetallic materials face the challenge of withstanding corrosive fluids and a wide temperature range. Water effects can be controlled through pH and dissolved salt adjustments, while oil effects depend on its composition [6]. Different types of polymers (PVC, amine, anhydride epoxy, PE, HDPE, PEX, PA11, PVDF, etc.) are used for pipes in various parts of oil facilities based on their temperature range. Polymer-based and other nonmetallic pipes offer corrosion and chemical resistance, reduced weight, and fewer deposits. However, they have limitations concerning temperature, pressure, mechanical strength, compatibility with hydrocarbons, UV exposure, environmental exposure, and joining and repair [7].

Some studies have been conducted on the use of nonmetallic pipes in oil and gas drilling. Barboza et al. [8] discussed a field experiment conducted in Venezuela using a PVC pipeline to transport oil at pressures of up to 70 psi and temperatures below 90 °F, highlighting the potential for such applications. The study suggests that thermoplastic pipelines like PVC offer advantages such as corrosion resistance and lower costs in handling and installation compared with traditional metallic pipelines. Cheldi et al. [9] asserted that spoolable reinforced thermoplastic pipes offer significant advantages in the O&G industry, including resistance to corrosion and fatigue, as well as improved flow rates. Their paper describes the installation of spoolable reinforced thermoplastic pipes in Iraq and Italy, demonstrating their suitability for different conditions, such as high pressures and corrosive environments, and the potential for wider use in oil production is suggested. Chen et al. [10] explore the impact of joining methods on the fatigue and fracture behavior of high-density polyethylene (HDPE) pipes, which are commonly used in various corrosive applications. Comparing electrofusion and butt-fusion joining techniques, the study reveals that butt-fusion results in better stiffness and strength compared with electrofusion. The study by Huang et al. [11] compared steel and non-steel slotted screen pipes for use in coal-bed methane wells. The collapse and bending strengths of J55 steel and PVC non-metal screen pipes were evaluated by analyzing the effects of slot width and density on pipe strength. Qamar and Pervez [12] carried out the tensile performance assessment of large-diameter hard PVC pipes used in thermally assisted wells for heavy oil recovery. They discussed different pipe structures and grip mechanisms, and the suitability of PVC pipes for specific applications.

### 1.3. Current Work

Though the studies mentioned in the previous section describe the use of nonmetallic pipes in drilling applications, they address other issues: polymers (HDPE, spoolable reinforced thermoplastic) other than PVC, oil-carrying pipes rather than pipes for water transport, and testing under tensile loads rather than compressive loads. This is why the need for the current study was crucial for the oil and gas industry, as described in this section.

In some oilfields in Oman and other countries, the carbonate structure is very tight and contains many fractures [13,14]. However, the matrix has a high saturation of heavy oil. In order to recover this oil, steam is injected to mobilize the oil for production through dedicated wells [15]. Very large quantities of water are needed to enable this steam injection; typically 40,000 m^3^/day. This water is usually obtained from nearby sources, say, about 30–40 km from the oilfield. In some cases, these fields are water-bearing sandstone of a highly unconsolidated nature. Sand control [16] becomes essential, with carbon-steel screens generally being used. These experience rapid and severe corrosion due to highly oxygenated water. 

To avoid this problem, sand screens of a different material are being tried out. One of the alternative materials being considered is a newly developed high-strength PVC. In the case of these PVC screens used in an Omani field, 2 out of 16 wells have experienced screen collapse. To avoid such failures, the testing and analysis of these PVC pipes and screens is a necessary prerequisite. Once sufficient confidence is built with the PVC concept, this well design can be introduced worldwide for drill-site water, camp supply, reverse osmosis (R/O) and fracking unit supply, water flooding, and steam generation. For a rough economic breakdown, assume that there are 30 shallow water supply wells per year. A switch from carbon steel to PVC could save approximately USD 150–200k per well. This leads to an annual saving of USD 4.5–6.0 million, or USD 20–30 million over 5 years.

Over the last ten years or so, a research unit at the Mechanical Engineering Department at Sultan Qaboos University has designed and constructed test rigs/facilities and conducted a large number of tests for the local petroleum development authority and other regional clients for tubular expansion, the burst and collapse of pipes, elastomer performance and characterization, the longevity evaluation of swell packers, the strength evaluation of GRE composite pipes, etc. [17,18,19]. This group was therefore approached for the proposed testing scheme to obtain more information on the strength of the applied PVC material. A test facility was designed and constructed, together with appropriate jigs and fixtures, to determine the following strength parameters for both blank PVC casings and PVC screens: compressive strength, tensile strength, collapse strength, and anisotropy behavior. In short, the work reported here is an integrity assessment under compressive loading.

## 2. Materials and Methods

### 2.1. Test Scope and Conditions

**Figure 1:**
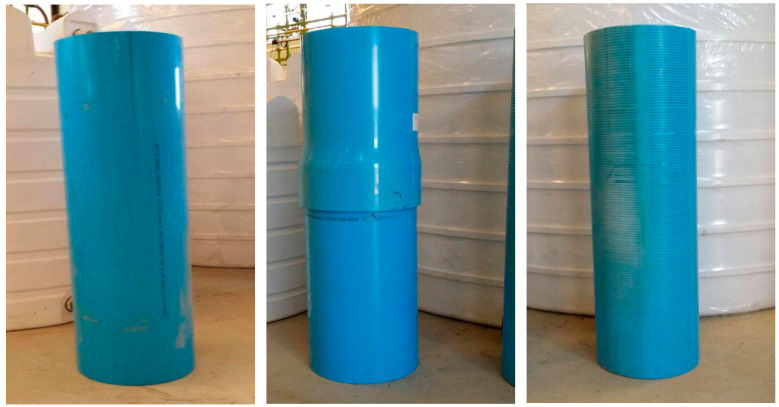
The three types of PVC pipes tested: plain, built-up, and slotted.

The main target of this study was the determination of the strength properties of high-strength PVC pipes under compressive loading by conducting experiments on actual pipes and the analysis of the test results. Three different sets of pipes were tested: plain pipes, built-up pipes (with a threaded connection in the middle), and slotted pipes (used as sand screens). The three pipe types are shown in Figure 1. As suggested by the field engineering team, the PVC pipes needed to undergo an aging procedure (3 months) before mechanical testing. Three pipes of each type (plain, built-up, and slotted) were tested to ensure the repeatability of the results. Without aging and with aging, this added up to testing 18 pipes. The dimensions of each pipe section were an outer diameter of 280 mm, a wall thickness of 240 mm, and a length of 1200 mm. The identification numbers and chemical composition of these pipes cannot be shared due to reasons of confidentiality.

### 2.2. Aging under Saline Water

**Figure 2:**
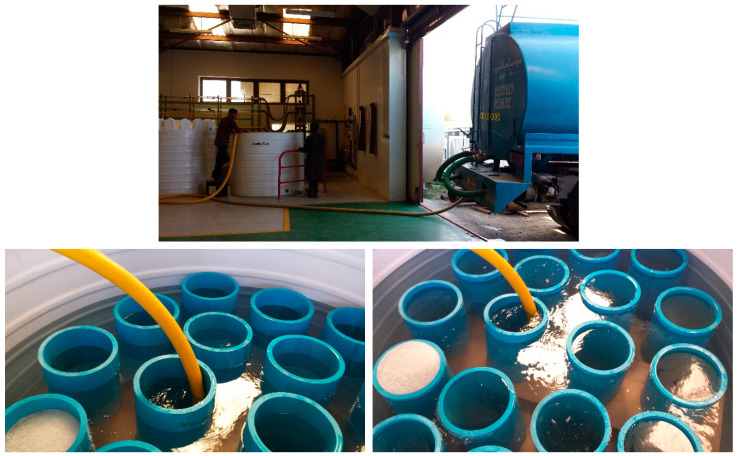
Immersion of PVC pipe sections in saline water for 3 months of aging.

As mentioned above, one requirement was to soak the compression test pipes in salt water with a prescribed salinity for 3 months before testing. This water was to have almost the same salinity as the field water (1.02 sg or 35 PPT). As each compression test was done 3 times, 9 PVC pipes were soaked (3 each of plain, built-up, and slotted types). This would need almost 2000 L of saline water (a little over 600 US gallons). To produce this amount of distilled water and then dissolve enough salt in it to obtain the required salinity would be a very costly task (time, equipment, effort, cost). After detailed discussions within the research group, a unique solution was identified. Our university has a desalination research unit near the sea, which can directly pump seawater through an underground tunnel and pumping system. Using a judicious mixing of normal drinking water and this high-salinity seawater, the required salt concentration could be achieved. However, special large-size containers needed to be arranged/constructed to keep these pipe sections submerged in this saline water (placing the 9 PVC pipes inside a 4000-gallon tank filled with saline water, with the help of a wooden holding fixture constructed in-house). Also, pumping this very large amount of seawater and transporting it from the desalination plant to the Engineering Research Lab (ERL) at the university was a daunting task. Figure 2 shows some images of the saline water transportation and filling work. 

### 2.3. Design and Construction of the Compression Test Setup

There is no standard test method for the strength measurement of large-diameter polymer (or PVC) pipes. Some examples of standards [20,21,22,23,24,25] that are not relevant for large-diameter PVC (or other polymer) pipes are the ASTM F2634–15 [20] Standard test method for laboratory testing of polyethylene (PE) butt fusion joints using tensile-impact method, ASTM F2928-18 [21] Standard practice for specimens and testing conditions for testing polyethylene (PE) pipe butt fusions using tensile and hydrostatic test method, ASTM E3102-18 [22] Standard practice for microwave examination of polyethylene electrofusion joints used in piping application, ASTM E3101-18 [23] Standard practice for microwave examination of polyethylene butt fusion joints, ASTM E3044/E3044M-16E1 [24] Standard practice for ultrasonic testing of polyethylene butt fusion joints, and ASTM E3167/E3167M-18 [25] Standard practice for conventional pulse-echo ultrasonic testing of polyethylene electrofusion joints. This is why the whole test setup was designed and constructed in-house, together with the necessary jigs and fixtures. 

**Figure 3:**
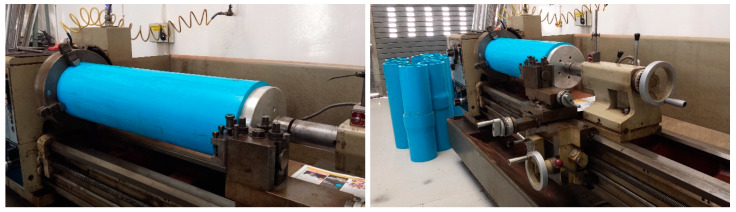
Facing operation being done on a lathe machine to guarantee flatness of the pipes on each end.

Compression tests were conducted on a Dartech universal testing machine with a capacity of 2000 kN in tension mode and 4000 kN in compression mode. It was observed that the pipe edges were not uniformly flat, as the 1.2 m pieces were cut out from 6 m PVC pipes. Without perfect flatness of the ends, the compression test would not remain correctly uniaxial. This means that the pipe sections could deform due to misalignment of the loading axis, and the test results may not be reliable. Therefore, the compression pipe sections went through precise edge trimming on a lathe machine to achieve flatness at both ends (Figure 3).

**Figure 4:**
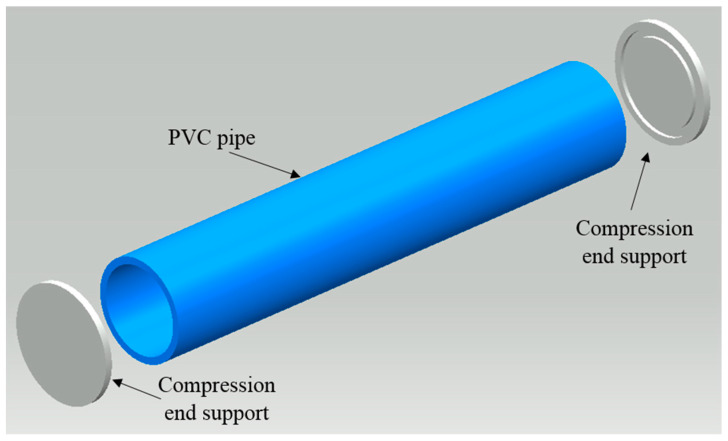
Compression test components: PVC pipe and two grooved end plates.

As the loads were expected to be quite high, there was an outside chance of the pipe slipping out of the compression chamber during testing. This would leave the test incomplete and would be a security risk. Two end plates (top and bottom) were therefore designed and constructed with a properly positioned groove to seat the PVC pipe (Figure 4). The diameter of these holding plates was about 30 cm more than the outer diameter of the pipe.

### 2.4. Compression Testing

**Figure 5:**
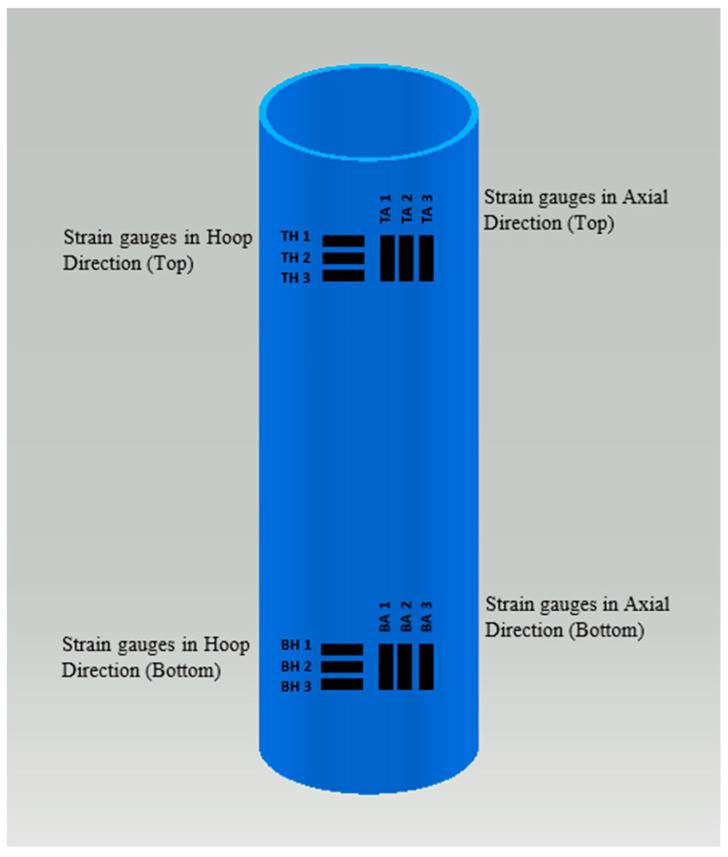
Sets of strain gauges in axial and hoop directions, near top and bottom ends of pipe.

**Figure 6:**
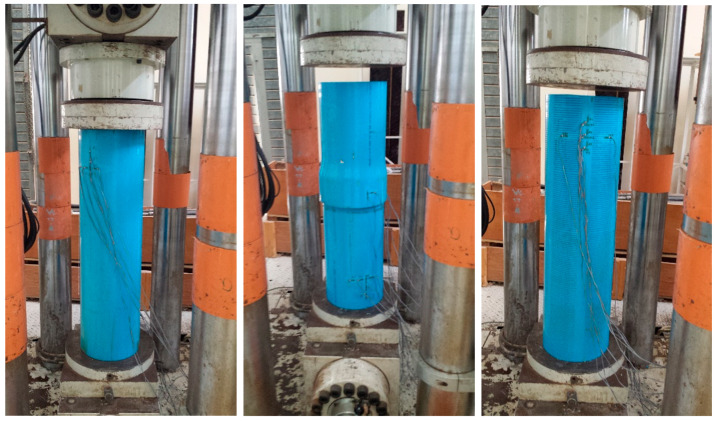
Plain, built-up, and slotted pipe loaded on the Dartec universal testing machine; strain gauges (fixed at prescribed distance from top and bottom ends) connected to data logger.

The loads (kN) and deformations (compression distance, mm) were directly recorded using the Dartech machine. With pipe dimensions measured before each test (length, outer diameter, thickness), machine readings could be converted into stress–strain data. However, for much more precise and complete data recording, strain gauges were used. Apart from the precision, another advantage of using strain gauges is that strains in the transverse direction can also be recorded, giving a measure for the anisotropy behavior (Poisson’s ratio) of the pipe material. As it was expected that deformations may be large (pipe material being a polymer), very special high-strain gauges (TML company) were procured especially for this project. The Dartech machine and all strain gauges were hooked up to a data logger and computer, which gave a real-time record of the loads and strains. Two sets of strain gauges (3 axial, 3 hoop) were fixed on each test pipe on the top and bottom positions on opposite sides of the pipe; in total, there were 12 gauges on each pipe. They were marked TA1 (top axial-1), BH1 (bottom hoop-1), etc. (Figure 5). These strain gages were positioned at a safe enough distance from the top and bottom grips to get rid of the end effect. To fix each strain gauge, careful grinding and partial polishing were done at the location on the pipe, and special glue was used to fix the gauge, which was followed by requisite curing. After attaching all 12 gauges, data cables were joined from each gage to the proper slot in the data logging setup, which was hooked up to the operating computer. Together with the end plates, the PVC pipe (with the strain gauge sets fixed and connected) was loaded into the Dartech machine, set to compression mode (Figure 6), and compressive loads were applied until fracture or buckling. 

## 3. Results

To ensure repeatability of the results, three tests were planned for each type of pipe (plain, built-up, slotted), with and without saline water aging. However, if the results from two tests almost matched, there was no need to go for the third test. Selected test result cases are shown below in pictorial and graphical formats. 

**Figure 7:**
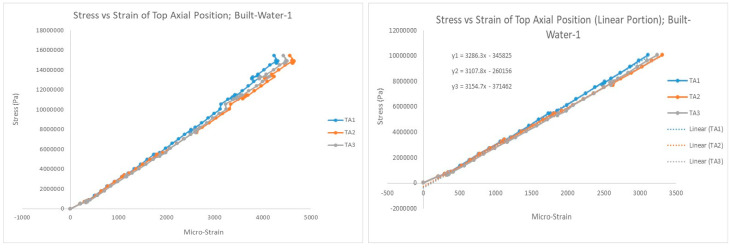
Sample stress–strain graph showing values from 3 strain gages and curve fitting for linear portion of the graph to extract elastic modulus (slope of line).

The system recorded force (kN), compression displacement (mm), and microstrain data for each test. These data sets were later converted into plots of stress vs. strain using average values of the pipe dimensions (length, outer diameter, thickness) measured before each test. Pipe properties were extracted from these graphs, such as fracture stress, fracture strain (or ductility), and Poisson’s number or anisotropy (ratio of hoop strain to axial strain). For the elasticity value (Young’s modulus) of the pipe, curve fitting was done only for the roughly linear portion of the graph, and the value of the slope of the fitted line was recorded. Figure 7 shows a sample graph (values from three strain gages), and its linear fitting. Note: microstrain values were used instead of strain. Microstrain (expressed as με) is a standard unit for special types of strain gauges and is described as a strain expressed in terms of parts per million [26]. 

**Figure 8:**
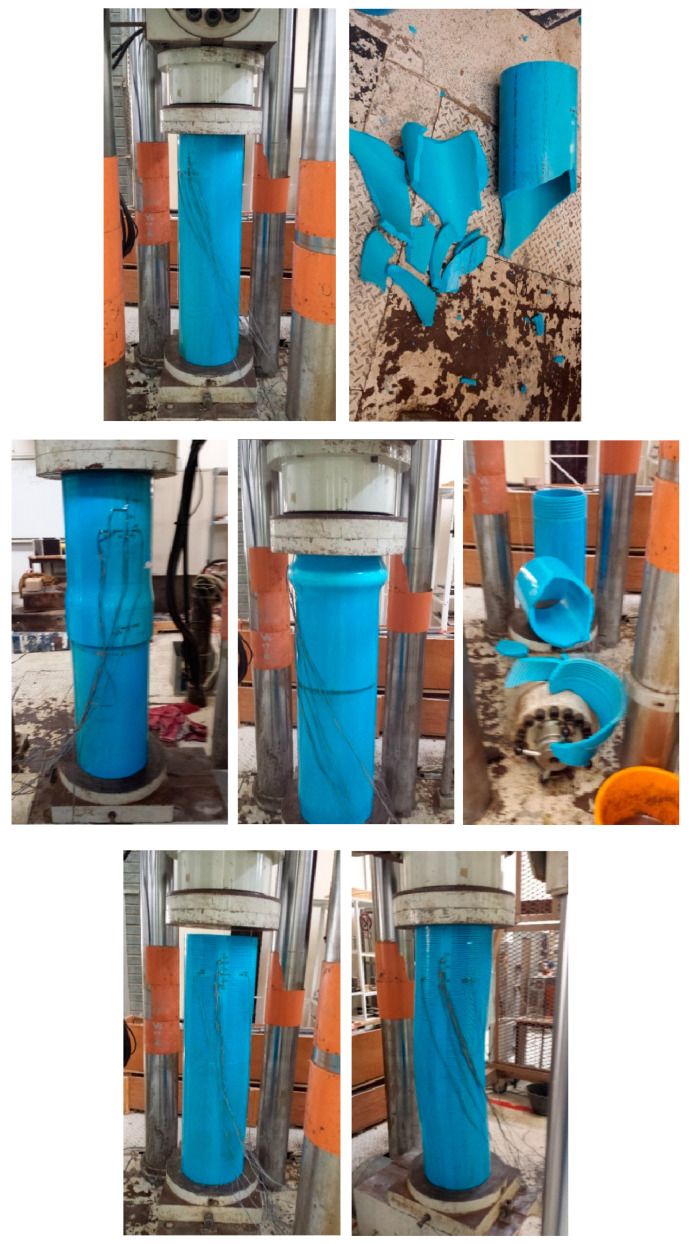
(**top**) Plain pipe before and after compression tests; it shattered into pieces during each test (**top**). (**middle**) Built-up pipe before and after compression tests; it shattered into pieces when load was applied until fracture and bulged at top when loading was stopped at this condition (**middle**). (**bottom**) Slotted pipe before and after compression tests; buckling-type curvature was clearly seen and would shatter into pieces if load was applied until fracture (**bottom**).

Figure 8 shows the PVC pipes before starting the compression test and at the end of the test. The plain pipe (Figure 8 top) fractured into pieces in each test, even after attempts at load control and displacement control to avoid explosive shattering. The built-up pipe (Figure 8 middle), with a threaded connection in the middle, shattered into pieces with continued compression but showed bulging at the top (without fracturing) when further loading was stopped after observing the bulge. The slotted pipe (Figure 8 bottom), obviously being the weakest of the three types, exhibited buckling-type failure, with significant axial curvature. If loading was not stopped at this stage, even these pipes would fracture into pieces. 

**Figure 9:**
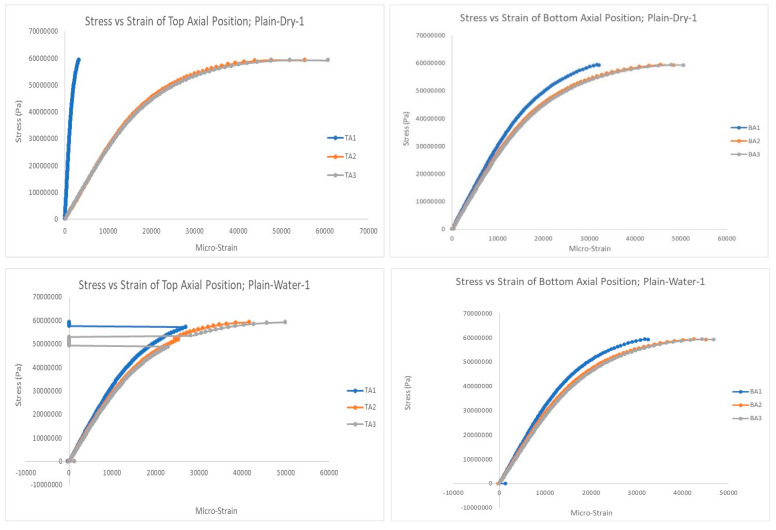
Stress–strain plots for plain pipe, top and bottom positions: before and after aging.

**Figure 10:**
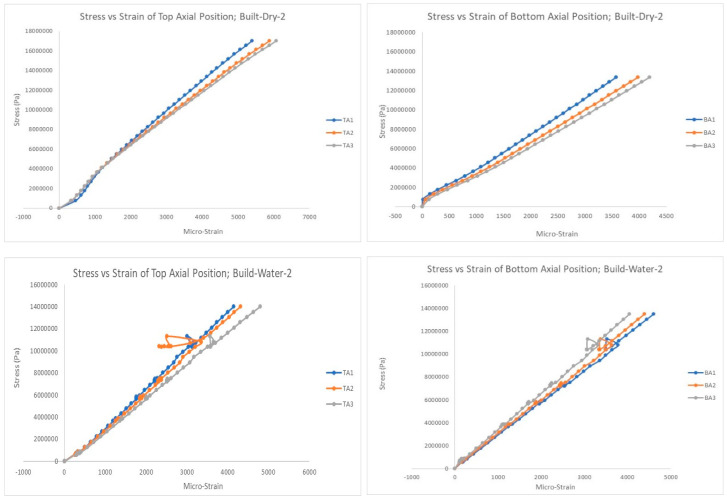
Stress–strain plots for built-up pipe, top and bottom positions: with and without aging.

**Figure 11:**
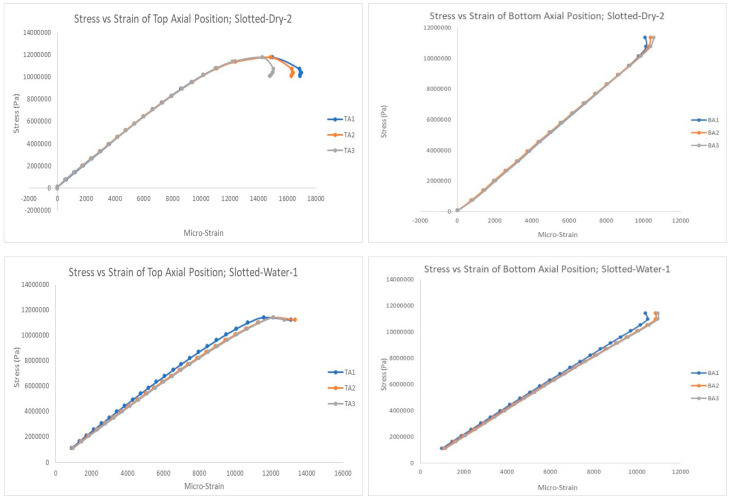
Stress–strain plots for slotted pipe, top and bottom positions: before and after aging.

Figure 9, Figure 10 and Figure 11 show selected stress–strain graphs of the compression tests of the three types of PVC pipes (plain, built-up, and slotted) in the as-is condition (without aging) and after aging (soaking) in saline water. As mentioned above, each test was conducted at least twice to ensure repeatability of the test results.

## 4. Discussion

Stress–strain data and graphs were used to extract pipe properties under compressive loading. Both ultimate strength and fracture stress values were extracted. However, they were consistently very close to each other, and thus, we denote both of them here by the single name of maximum stress. The average values of stiffness (elastic/Young’s modulus), maximum stress, ductility or maximum strain, and anisotropy behavior (Poisson’s ratio) are summarized in Table 1. More salient test results are discussed below for each pipe type and aging condition.

The stiffness of the built-up pipe was a little higher than the plain one, as expected. The built-up pipe had a significant threaded portion in the middle, adding major residual stresses to the pipe and increasing the stiffness. However, the highest values of the elastic modulus for the slotted pipes were quite surprising. The stiffness generally increased a little after soaking in saline water, with a much larger variation for slotted pipes. The values were confirmed through repeated tests. One possible reason for the anomalous observation may be that the value of elastic modulus was determined by considering only the linear portion of the stress–strain curve and fitting a straight line through these data points. These are polymer pipes, and no portion of the stress–strain diagram was really linear; therefore, this straight-line fit was rather approximate.

As expected, the maximum stress that these PVC pipes could withstand before compressive failure reduced a little (11 to 12%) after soaking in saline water. However, the built-up and slotted pipes had significantly lower strength than the plain PVC pipes. The reason for the lower strength of the built-up pipes was the same; large amounts of residual stresses due to the threaded joint between the two pipe sections. The slotted pipes were of course the weakest because of the lack of material due to the large number of slots, which also provided major stress concentration points. The maximum allowable water pressure should be limited (with a reasonable factor of safety) based on the weakest link in terms of pipe strength (slotted pipe).

The highest value of pipe ductility (maximum strain %) was for the plain pipe, while the lowest was for the built-up type. Added stresses due to the threaded portion significantly reduced the pipe flexibility, leading to a reduced amount of total deformation (or strain) under compression. The ductility was generally reduced a little after aging, but not to any drastic degree.

Poisson’s ratio for all three pipe types (plain, built-up, and slotted) and under both conditions (before and after aging) remained approximately in the 0.43–0.45 range. This indicates that the increase in pipe diameter (deformation in the hoop direction) would be roughly 43% to 45% of the reduction in pipe length (deformation in the axial direction) under compressive loading. This near-constant value of anisotropy is very encouraging, both for field engineers and for future attempts at numerical simulation of these pipes for different water fields.

The primary takeaway from all experimental results is that it is possible to effectively substitute corrosion-vulnerable steel pipes and sand screens with corrosion-resistant, high-strength PVC pipes and screens for the purpose of transporting water in thermal wells. Another very encouraging observation for the field engineers is that there was an almost insignificant effect of aging on the major compressive properties (maximum stress, maximum strain, and Poisson’s ratio) of all three pipe types. This means that all structural design and related calculations and analyses can be based on as-produced pipes, without going through the complex 3-month aging process. 

## 5. Conclusions

Steam injection is used to enhance the flow of heavy oil in high-viscosity wells. This study addressed the challenges of water requirements in steam generation plants and corrosion issues in steel screens used for sand control in fragmented water reservoirs. To combat corrosion losses, this study explored the use of non-metallic materials, particularly novel high-strength PVC pipes. The research focused on assessing the structural integrity of large-diameter hard PVC pipes under compressive loading. The pipes were subjected to a 3-month aging process in saline water to mimic real field conditions. Dynamic testing was conducted using strain gauge sets to measure longitudinal and transverse deformations. The results provide valuable insights for field engineers to select appropriate pipes for specific oilfields.

Stress–strain data and graphs were utilized to analyze pipe properties under compressive loading. Average values of stiffness, maximum stress, ductility (maximum strain), and anisotropy behavior (Poisson’s ratio) were determined and summarized. The results showed that the built-up pipe had slightly higher stiffness due to residual stresses from the threaded portion. Surprisingly, slotted pipes exhibited the highest elastic modulus. After aging in saline water, the stiffness of all pipes increased, especially for slotted pipes. The maximum stress decreased by 11–12% after aging, and built-up and slotted pipes had lower strength than plain PVC pipes due to residual stresses and material reduction, respectively. The ductility was the highest for plain pipes and the lowest for built-up pipes. Poisson’s ratio remained consistent for all pipes and aging conditions. Fortunately, aging had an insignificant effect on major compressive properties, allowing engineers to base structural design calculations on as-produced pipes without the need for a 3-month aging process. The key finding is that it is very feasible to substitute corrosion-susceptible steel pipes and sand screens with corrosion-resistant, high-strength PVC pipes and screens in the context of water transport for steam injection and other thermal wells.

## Figures and Tables

**Table 1:** Average values of compression test results for the three pipe types, with and without aging (mean ± SD).

Pipe Type/Condition	Stiffness (Elastic Modulus) GPa	Max Stress (MPa)	Maximum Strain (%)	Anisotropy (Poisson’s Ratio)
Plain (as is)	2.93 ± 0.23	62.02 ± 1.898	3.87 ± 0.001	0.43 ± 0.03
Plain (after aging)	3.24 ± 0.19	54.1 ± 2.67	2.44 ± 0.027	0.44 ± 0.03
Built-up (as is)	3.04 ± 0.27	17 ± 1.23	0.52 ± 0.015	0.44 ± 0.04
Built-up (after aging)	3.34 ± 0.33	15.4 ± 1.35	0.45 ± 0.02	0.42 ± 0.06
Slotted (as is)	1.05 ± 0.01	11.76 ± 0.69	1.04 ± 0.002	0.45 ± 0.07
Slotted (after aging)	5.76 ± 0.39	11.44 ± 0.11	1.51 ± 0.003	0.47 ± 0.03

## Data Availability

Not available.

## Nomenclature

PVC	Polyvinyl chloride
PE	Polyethylene
HDPE	High-density polyethylene (HDPE)
PEX	Cross-linked polyethylene
PA11	Nylon 11 or polyamide 11
PVDF	Polyvinylidene fluoride or polyvinylidene difluoride (PVDF)
PPT	Parts per trillion; PPT = PPM/1000
PPM	Parts per million
UV	Ultraviolet
GRE	Glass-reinforced epoxy (composite)
SD	Standard deviation
